# How to Undertake Personalized Assessments and Administer Cures for Pain

**DOI:** 10.3390/jpm15120630

**Published:** 2025-12-18

**Authors:** Barbara Rocca

**Affiliations:** Children’s Orthopedics, Lenval Hospital, 57, Av. Californie, 06200 Nice, France; barbararocca81@gmail.com

## 1. Introduction

Pain is a multidimensional and highly individualized experience shaped by biological, psychological, and social determinants. Advances in pain science over the last decade have increasingly emphasized the importance of personalized approaches to both assessment and treatment. Rather than viewing pain as a purely biomedical phenomenon, contemporary research highlights its complex interplay with cognitive, emotional, behavioral, and environmental factors. This Special Issue gathers six contributions that explore these dimensions and illustrate how personalization can enhance understanding, management, and outcomes in patients experiencing acute, chronic, or postsurgical pain.

## 2. Recent Developments in Personalized Pain Assessment

Personalized evaluation has evolved significantly with the integration of psychological profiling, patient-reported outcome measures (PROMs), neurophysiological techniques, and biopsychosocial screening. Recent evidence demonstrates that psychological traits—such as emotional stability, coping strategies, and personality dimensions—meaningfully influence pain perception, disability, and quality of life (contribution 1).

Sex- and gender-related differences have also emerged as critical determinants of pain trajectories. Biological, psychosocial, and sociocultural variables influence differential pain responses and disability patterns between men and women (contribution 2).

Additionally, advancements in measurement tools have strengthened personalized assessment. Validated short-form PROMs, such as updated versions of the Roland–Morris Disability Questionnaire, allow clinicians to monitor disability efficiently and reliably in daily practice (contribution 3).

## 3. Innovations in Personalized Pain Therapies

The shift toward personalization has catalyzed therapeutic innovation. Non-invasive brain stimulation (NIBS), particularly cerebellar stimulation, represents a promising frontier for chronic pain treatment. The cerebellum plays a key role in sensory, affective, and cognitive modulation of pain, and early evidence suggests beneficial effects of cerebellar-targeted stimulation (contribution 4).

Patient-centered care models are increasingly integrated into pain management. Shared decision-making has demonstrated significant promise in improving understanding, empowerment, satisfaction, and adherence among patients with acute neuropathic pain such as postherpetic neuralgia (contribution 6) [1,2].

Despite theoretical support, structural barriers limit the clinical implementation of the biopsychosocial model. Insufficient training, organizational constraints, and limited time remain key challenges for clinicians (contribution 5).

## 4. Integrating Insights Across Contributions

Together, the contributions emphasize that personalized pain care requires multidimensional evaluation and tailored interventions. They collectively show the following:

- Psychological traits affect long-term surgical outcomes (contribution 1).
- Sex-specific differences shape chronic pain trajectories (contribution 2).
- Neurophysiological innovations such as cerebellar NIBS offer promising personalized interventions (contribution 4).
- Efficient, validated PROMs support accessible, patient-centered assessment (contribution 3).
- Systemic improvements are essential for biopsychosocial implementation (contribution 5).
- Shared decision-making enhances treatment engagement and outcomes (contribution 6).

## 5. Future Directions

Future research should prioritize the following points:

1. Refined psychological profiling integrating biological and emotional markers.
2. Advanced understanding of sex- and gender-based mechanisms influencing pain.
3. Development of neurophysiological biomarkers for personalized neuromodulation.
4. Implementation studies to overcome barriers to the biopsychosocial model.
5. Digital PROMs and remote monitoring for continuous assessment.
6. Structured shared decision-making tools across different pain populations.
7. Multimodal therapeutic approaches combining psychological, physical, and neurophysiological interventions.

## 6. Conclusions

This Special Issue provides a comprehensive overview of the current state of personalized pain science, highlighting its clinical, psychological, and neurophysiological dimensions. The six contributions collectively affirm that effective pain management must consider individual differences across biological, emotional, cognitive, and social domains. By advancing assessment tools, treatment strategies, and implementation frameworks, this Special Issue supports continued progress toward truly personalized pain medicine [3,4,5,6,7,8,9,10].
